# Regulation of solvent tolerance in *Pseudomonas putida* S12 mediated by mobile elements

**DOI:** 10.1111/1751-7915.12495

**Published:** 2017-04-11

**Authors:** Rohola Hosseini, Jannis Kuepper, Sebastian Koebbing, Lars M. Blank, Nick Wierckx, Johannes H. de Winde

**Affiliations:** ^1^ Microbial Biotechnology and Health Institute of Biology Leiden University Leiden The Netherlands; ^2^ Institute of Applied Microbiology – iAMB Aachen Biology and Biotechnology – ABBt RWTH Aachen University Aachen Germany

## Abstract

Organic solvent‐tolerant bacteria are outstanding and versatile hosts for the bio‐based production of a broad range of generally toxic aromatic compounds. The energetically costly solvent tolerance mechanisms are subject to multiple levels of regulation, involving among other mobile genetic elements. The genome of the solvent‐tolerant *Pseudomonas putida* S12 contains many such mobile elements that play a major role in the regulation and adaptation to various stress conditions, including the regulation of expression of the solvent efflux pump SrpABC. We recently sequenced the genome of *P. putida* S12. Detailed annotation identified a threefold higher copy number of the mobile element ISS12 in contrast to earlier observations. In this study, we describe the mobile genetic elements and elaborate on the role of ISS12 in the establishment and maintenance of solvent tolerance in *P. putida*. We identified three different variants of ISS12 of which a single variant exhibits a high translocation rate. One copy of this variant caused a loss of solvent tolerance in the sequenced strain by disruption of *srpA*. Solvent tolerance could be restored by applying selective pressure, leading to a clean excision of the mobile element.

## Introduction

### *P. putida* S12

*Pseudomonas putida* S12 is a Gram‐negative bacterium that was isolated on styrene as a sole carbon source (Hartmans *et al*., 1990). It has the exceptional ability to tolerate a wide variety of toxic organic solvents in concentrations that are lethal to most microorganisms (Isken and de Bont, 1998). *P. putida* S12 is able to metabolize organic solvents such as styrene or 1‐octanol and is also highly resistant towards non‐metabolized organic solvents such as toluene. This ability is extremely beneficial for the production of fine chemicals and their removal using two‐phase water‐organic solvent fermentation systems (Wierckx *et al*., 2005; Heipieper *et al*., 2007; Verhoef *et al*., 2009). As such, *P. putida* S12 is a promising platform for the production of aromatic compounds (Nijkamp *et al*., 2005; Wierckx *et al*., 2005; Tiso *et al*., 2014; Loeschcke and Thies, 2015).

### Solvents and their effects on bacterial membranes

Organic solvents of a log P_OW_ of 1–4 are destructive for bacterial lipid membranes (Heipieper *et al*., 2007). These solvents accumulate in lipid membranes, causing an increase in membrane fluidity and a decrease in bilayer stability (Sikkema *et al*., 1994; Weber *et al*., 1994). Loss in bilayer stability contributes to a passive flux of protons across the membranes and affects the ATP synthesis by the proton motive force PMF. In addition, the membrane protein interactions are altered due to this unstable lipid bilayer (Sikkema *et al*., 1994). To compensate for these adverse conditions, solvent‐tolerant *P. putida* strains adapt to these conditions by changes in their inner‐ and outermembranes and by the activation of an active efflux pump for organic solvents (Heipieper and de Bont, 1994; Weber *et al*., 1994; Ramos *et al*., 1997; Isken and de Bont, 1998; Ramos *et al*., 2002; Ramos *et al*., 2015). The solvent efflux pump SrpABC in *P. putida* S12 is exclusively activated by solvent stress (Kieboom *et al*., 1998b; Kieboom and de Bont, 2001). Highly similar efflux pumps have also been identified in other solvent‐tolerant strains, such as TtgGHI in *P. putida* DOT‐T1E (Segura *et al*., 2003).

### Insertion sequence‐mediated regulation

Insertion sequences are the smallest transposable elements in prokaryotes with extensive effects on genome plasticity (Casacuberta and González, 2013). They have strong relevance regarding gene (in)activation and thus play an important role in adaptive responses of microorganisms to environmental changes and challenges. Phenotypic variations within a population have been recognized as traits that enable survival or adaptation to specific environments (van der Woude and Bäumler, 2004). These variations have been mainly studied in the context of host–pathogen interactions, although in *P. putida* and *P. brassicacearum* phase, variation is also involved in the production of exo‐enzymes and secondary metabolites (Chabeaud *et al*., 2001; van den Broek *et al*., 2003). Transposition of insertion sequences has been associated with phase variation, affecting the cell surface morphology in *N. meningitides* and *S. epidermidis* (Ziebuhr *et al*., 1997; Hammerschmidt *et al*., 1996). Examples of insertion sequences that cause a specific phenotype in *P. putida* S12 are the ISS12 and the ISPpu21. Both insertion sequences were found to be present in the *srpS* repressor gene of the solvent efflux pump SrpABC in a subpopulation of *P. putida* S12, thus increasing the constitutive expression of the solvent resistance pump and consequently the survival upon solvent stress (Wery *et al*., 2001; Sun and Dennis, 2009).

### Insertion sequences in the *P**. **putida* S12 genome

The complete genome of *P. putida* S12 has been recently sequenced and was found to consist of a circular chromosome (5799 Kbp) and a megaplasmid 584 Kbps (Kuepper *et al*., 2015). The chromosome has an average GC content of 61.8%, and the megaplasmid has a 57.8% GC content [accession no: CP009974 (chromosome), CP009975 (plasmid pTTS12)]. Surprisingly, this genome showed a high number of one specific insertion sequence, ISS12, while in the previous studies, fewer ISS12 copy numbers were observed in *P. putida* S12 using Southern blot analysis (Wery *et al*., 2001; Volkers *et al*., 2010).

Investigating the high occurrence of ISS12 mobile element in the recently sequenced *P. putida* S12 genome (here after referred to as *P. putida* S12/A) led to an interesting observation. To our surprise, the *P. putida* S12/A could not tolerate a second phase of styrene or sudden solvent shock of toluene as reported for the original strain. In this study, the role of insertion sequences (specifically ISS12) in the regulation of solvent tolerance was investigated. We compared the *P. putida* S12/A to its original stocks of *P. putida* S12 currently available at TNO (the Netherlands) and the ATCC collection (USA).

## Results and discussion

### Mobile elements in *P. putida* S12

Detailed annotation of mobile elements was performed on the sequence of *P. putida* S12, to confirm the high occurrence of mobile elements in the *P. putida* S12/A. An overview of characterized mobile elements in *P. putida* S12/A is summarized in Table 1. This strain contains five Tn3‐like and two Tn7‐like transposable elements. However, the majority (~55 copies) of mobile elements are characterized as insertion sequences that belong to different families, such as IS3, IS5 and IS21. These sequences showed highest DNA similarity to other insertion sequences of *Pseudomonas* strains, using BLAST search and comparison with the ISFinder database (Siguier *et al*., 2005). The IS3, IS66 and IS110 family of insertion sequences (~11 copies) were observed exclusively on the chromosome, while IS256, IS110‐IS1111, IS5‐IS427 and Tn3‐like (~eight copies) were only observed on the megaplasmid. The remaining insertion sequences, ISS12 and ISPpu21, were observed on both the chromosome and the megaplasmid.

**Table 1:** Mobilome of *P. putida* S12

Name	Family group	Homologous sequences	Identity	Copy number	Location
IS66	IS66	ISPpu15	83%	2	Chromosome
ISPpu21	IS5	ISPpu21	100%	4	Chromosome/plasmid
IS3	IS3	ISPpu22	92%	1	Chromosome
ISS110	ISS110	ISPpu9	85%	4	Chromosome
IS256	IS256	ISXax1	79%	1	Plasmid
IS3‐IS150	IS3‐IS150	ISPsy8	69%	5	Chromosome
Tn3‐like	Tn3	ISXc4	68%	2	Plasmid
Tn3‐like	Tn3	TnAs1	92%	2	Plasmid
Tn7‐like	Tn7	Tn7‐like	100%	2	Chromosome/plasmid
Tn3‐like	Tn3	TnAs3	77%	1	Plasmid
IS110‐IS1111	IS110‐IS1111	ISAzvi2_aa1	75%	1	Plasmid
IS5‐IS427	IS5‐IS427	ISPst8	77%	1	Plasmid
ISS12	IS21	ISS12 ISPpu7	100%	28	Chromosome/Plasmid
ISS12_C	IS21	ISPsy14	98%	4	Chromosome/Plasmid
ISS12_D	IS21	ISPsy14	85%	1	Chromosome/Plasmid
Unidentified				18	Chromosome/plasmid

We focused on ISS12 insertion sequences, because the translocation of this element has been linked to resistance and adaptation of *P. putida* towards solvents (Wery *et al*., 2001; Volkers *et al*., 2010). ISS12 can translocate into the repressor *srpS* of the *srpABC* operon, which bestows upon *P. putida* S12 its characteristic solvent tolerance (Kieboom *et al*., 1998a; Kieboom and de Bont, 2001; Volkers *et al*., 2015). ISS12‐mediated disruption of *srpS* leads to constitutive expression of *srpABC*, which enables survival of a small subpopulation upon a sudden solvent shock (Wery *et al*., 2001). Additional translocations of ISS12 were observed after adaptation of *P. putida* S12 to high concentrations of benzene (Volkers *et al*., 2010). *P. putida* S12/A showed a threefold higher copy number of the insertion sequence ISS12 in contrast to earlier observations based on Southern blot data (Wery *et al*., 2001; Volkers *et al*., 2010).

### ISS12 genetic diversity and organization

With 33 copies, ISS12 elements comprise half of the total insertion sequences in the *P. putida* S12/A analysis. BLAST search reveals several matches with 70–99% DNA sequence identity to other insertion sequences in *Pseudomonas* strains (Fig. S1). These include other *P. putida* strains as well as *P. pseudoalcaligenes*,* P. aeruginosa, P. mandelii* and *P. stutzeri*. Other insertion sequences were observed with < 70% similarity, mainly in *Marinobacter, Burkholderia* and *Polaromonas* species.

**Figure 1:**
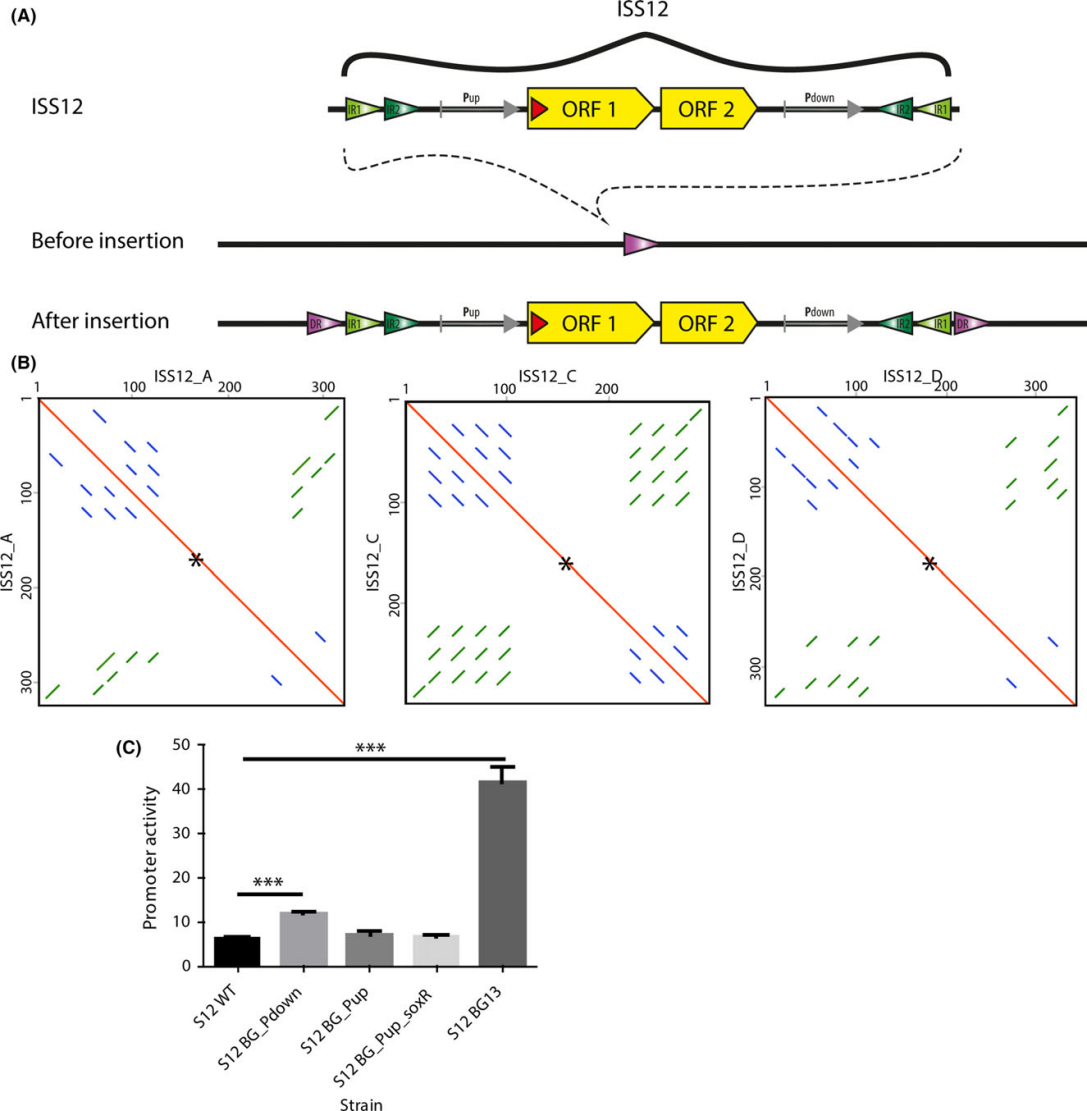
Insertion sequence ISS12 and its variants in *P. putida* S12. A. The ISS12 sequence contains two open‐reading frames (ORFs) and two inverted repeats at the flanks (IR). After insertion in a DNA sequence, a directed repeat (DR) is observed at the flanks of ISS12 that has been created during the insertion. B. Self‐plot of ISS12‐variants without ORF1 and ORF2; the black asterisks (*) on red line represent the location of ORFs. The self‐plot shows different variations in inverted repeats (green) and directed repeats (blue) within the ISS12‐variants. C. Activity of ISS12‐putative promoter sequences integrated in *P. putida* S12 using Tn7 probe vector. The promoter activity was calculated based on GFP expression of promoter‐GFP constructs for the P_up_, P_up_soxR_, P_down_ (indicated in A) and compared to background fluorescence signal of *P. putida* S12 and the positive control integrated BG13 promoter (P_em7_‐BCD2). The mean of triplicates is shown in the graph, and error bars indicate standard deviation. ***indicates *p*‐value < 0.001 with unpaired student *t*‐test.

ISS12 consists of 2596 bp and is one of the largest insertion sequences. It contains two open‐reading frames (ORFs), encoding an integrase that enables the transposition and an ATPase AAA helper protein (Fig. 1). The ORFs are flanked by ±150‐bp sequence containing inverted repeat regions (Fig. 1A). The majority of ISS12 elements (28 copies) have a 100% similarity to the previously described sequence of ISS12 (accession number AF292393, Wery *et al*., 2001). Within ISS12, two putative regulatory sequences were proposed, one at the upstream of transposase (P_up_) and one at the downstream of the ATPase AAA encoding gene (P_down_) (Wery *et al*., 2001; indicated in Fig. 1B). A putative SoxR binding motif (Palma *et al*., 2005) lies just downstream of P_up_, 25 base pairs after the ATG start site of transposase encoding gene (indicated in Fig. 1A and Fig. S2). The P_up_, P_up_ including SoxR motif (P_up_soxR_) and P_down_ promoter sequences were cloned in a Tn7 promoter‐probe vector (Zobel *et al*., 2015) and integrated into *att*Tn7 site in *P. putida* S12. The analysed promoter activity of P_up_ and P_up_soxR_ was similar to the background autofluorescence signal of the negative control S12 WT (Fig. 1C). Thus, surprisingly, these putative upstream promoters are inactive under the tested conditions, indicating that the transposase must be activated either by upstream regulatory elements, or that some other form of transcriptional regulation is imposed (Reimmann *et al*., 1989). The downstream promoter P_down_ has a relatively low activity, which is approximately sixfold weaker than P_em7_ in *P. putida* S12 (Fig. 1C), a synthetic promoter of average strength (Zobel *et al*., 2015). No effect was observed on promoter activities under oxidative stress conditions using 0.25 and 0.5 mM H_2_O_2_ (Fig. S3), indicating that the putative SoxR binding motif is not involved.

We identified two other variants of the insertion sequence after alignment of all ISS12 insertion sequences. According to our analysis, all ISS12 variants were already present in previous sequences of *P. putida* S12 and are not the result of mutations during transposition in S12/A strain. These variants were named ISS12_C and ISS12_D with 79% and 76% identity to ISS12 respectively (Table 2 and Fig. 1). The ISS12 sequence (at position 5324675–5327253 on CP009974) that previously was annotated as ISS12_B had 100% identity to ISS12_C sequence. This became apparent after cloning all ISS12 variants of *P. putida* S12 in pJET1.2 and sequencing (Fig. S4). This was probably caused during the assembly process at that specific region due to high occurrence of this insertion sequence. The ISS12_C variant has four copies in the *P. putida* S12/A genome, and the reading frames of the two encoded genes and start/stop codons are retained. The encoded proteins of the ISS12_C variant show an 84% and 85% identity at the amino acid level to the integrase and *ATPaseAAA* encoding genes of ISS12. The inverted repeats (IR) are different although there are still several candidate IRs present in this ISS12 variant (Fig. 1B). Inverted repeat regions at the flanks of insertion sequences play an important role in their translocation efficiency. They often contain the transposase binding site as well as inverted repeats for DNA secondary structures required for transposition (Reimmann *et al*., 1989; Berger and Haas, 2001). The ISS12_C is also an active insertion sequence, which was demonstrated by two new translocation events into the chromosome of *P. putida* S12/A. It has highest similarity to the ISPsy14 with 98% identity based on the amino acid sequence of two ORFs. The ISS12_D variant is only present in a single copy and has a frameshift in the open‐reading frames of the integrase encoding genes, whereas the *ATPaseAAA* gene is retained normally. The encoded *ATPaseAAA* gene of the ISS12_D variant shows a 76% identity at the amino acid level to the *ATPaseAAA* in ISS12. This variant also shows candidates for alternative inverted repeats, and it has an additional 50 bp at the 5′ prime region (Fig. S2). The single occurrence of this ISS12 variant indicated that it might be a silent insertion sequence due to an early stop codon in ORF1 and a larger repeat region at its left flank compared to other ISS12 variants. Similar as ISS12_C, ISS12_D has the highest (~85% based on amino acid sequence) similarity to the ISPsy14. ISS12_A appears to exhibit a high translocation rate in *P. putida* S12/A with 28 copies.

**Table 2:** Similarity of different ISS12 variants in *P. putida* S12 compared to AF292393

ISS12 variants	ORF 1^a^	ORF 2^a^	IR1	IR2	DR	Copy number
ISS12_A	100%	100%	100%	100%	Variable	28
ISS12_B	96%	100%	86%	78%	Variable	1
ISS12_C	84%	84%	86%	78%	Variable	3
ISS12_D	76%	85%	86%	89%	Variable	1

IR1 and IR2 are the inverted repeats at the flanks, and DR is the directed repeat sequence outside the flanks of ISS12.

a ORF1 and ORF2 sequence similarity is based on the amino acid sequence.

### *P. putida* S12: A genome in motion

To investigate the high occurrence of ISS12 in the sequenced *P. putida* S12, a detailed comparison was performed with previously available sequencing data (Table 3). The older shotgun sequencing (accession number: AYKV00000001) and two unpublished sequences of phenol‐ and cinnamic acid‐producing mutants (Nijkamp *et al*., 2005 and Wierckx *et al*., 2005) were re‐assembled using the complete sequence as the backbone. The assembly parameters were set as such to avoid assembly of reads that would match to multiple regions in the backbone. The presence of ISS12 in re‐assembled data of previous sequenced S12 strains could be observed clearly by a successful alignment of reads at the 5′ and 3′ flanks of insertion elements, while ISS12s unique to the S12/A strain showed clear gaps in the re‐assembly data (Fig. S5). The ISS12 elements that were not present in previously published sequences of *P. putida* S12 were removed from the backbone, and by repeating the assembly process, we observed complete coverage at those specific positions. Of 33 insertion sequences, 23 are unique to the *P. putida* S12/A sequenced in 2015. Table 3 gives an overview of the location of the insertion sequences and their absence or presence in the other three *P. putida* S12 genome sequences. To validate the sequence analysis on the presence and absence of the ISS12 in the original *P. putida* S12 (freshly acquired from ATCC, 700801), we performed PCRs on 33 regions in both the original and the recently sequenced strains. Ten sequence regions of ISS12 showed a PCR product of 3 kb in the original strain, while the remaining regions showed a PCR product of 400 bp and thus no ISS12 insertions, as expected (Table 3). In the recently sequenced strain, all PCR products showed the expected 3‐kb product of ISS12.

**Table 3:** Analysis of ISS12 insertion sites in *P. putida* S12/A

Located on	Variant	Position	Orientation	Presence in previous sequences	Directed Repeat (sequence)	Directed Repeat (length)	ISS12_location
Chromosome	ISS12	5798528‐2596	Reverse	No	GGAAGTG	7	Integrase – Peptidase S24^b^
Chromosome	ISS12	1303416‐1306017	Forward	No	TCTGTG	6	Membrane protein (UPF0114 family) – cation transporter^b^
Chromosome	ISS12_C	1343201‐1345781	Forward	No	CACTACC	7	Putative Holin – putative integrase^b^
Chromosome	ISS12	1508754‐1511356	Forward	No	CGCCGAC	7	Major facilitator transporter (MFS)^a^
Chromosome	ISS12	1901500‐1904102	Forward	No	TTCTTCG	7	ABC transporter^a^
Chromosome	ISS12	2092282‐2094884	Forward	Yes	GCAAAAC	7	Copper resistance gene^a^
Chromosome	ISS12	2509647‐2512248	Forward	No	CAACAT	6	Hypothetical protein^a^
Chromosome	ISS12	2747110‐2749712	Forward	No	GCATAGC	7	Amidohydrolase, SalR regulator^a^
Chromosome	ISS12	2756345‐2758947	Reverse	No	GACGGTT	7	Benzoylformate decarboxylase^a^
Chromosome	ISS12	2767364‐2769967	Reverse	No	CTCGTATG	8	Serine – tRNA ligase^c^
Chromosome	ISS12	2913093‐2915695	Reverse	No	CCATGAC	7	Hypothetical (First gene in Type IV secretion operon)^a^
Chromosome	ISS12	3486765‐3489368	Forward	No	CACAGGGA	8	PrrB RsmZ‐like ncRNA^a^
Chromosome	ISS12	3503916‐3506518	Reverse	No	CGAGGTG	7	TRNA 2‐thiocytidine biosynthesis protein^c^
Chromosome	ISS12	4425759‐4428360	Forward	No	CGGGGG	6	Glutaminase^a^
Chromosome	ISS12	4894007‐4896609	Reverse	Yes	CGCAAGT	7	Glycosyl hydrolase family 32^a^
Chromosome	ISS12	4905864‐4908466	Forward	No	AGCTCGA	7	Hypothetical protein^a^
Chromosome	ISS12_D	4954933‐4957427	Forward	Yes	ATGACA	6	Hypothetical^c^
Chromosome	ISS12	4963941‐4966543	Forward	Yes	GACAAGC	7	SH3 domain‐containing protein^a^
Chromosome	ISS12	5273052‐5275654	Reverse	No	CATAGGC	7	GntR transcriptional regulator – Transporter^a^
Chromosome	ISS12_C	5324675‐5327253	Forward	No	GGCAGTC	7	Transposase^c^
Chromosome	ISS12	5641776‐5644378	Forward	Yes	CTTGATG	7	Major facilitator transporter (MFS)^a^
Plasmid	ISS12_C	53788‐56369	Reverse	Yes	GCCCTGCC	8	Transposase – Hypothetical protein^b^
Plasmid	ISS12	80378‐82980	Forward	No	TCTACAC	7	Tellurium resistance‐like protein^a^
Plasmid	ISS12	115546‐118148	Forward	No	CGTAGGG	7	Hypothetical protein^a^
Plasmid	ISS12	200772‐203372	Forward	Yes	CCTTGCC	7	Hypothetical protein^a^
Plasmid	ISS12	218063‐219257	Forward	No	CCGCT	5	Hypothetical protein^a^
Plasmid	ISS12	240098‐242700	Forward	Yes	ACCAACC	7	Hypothetical protein^a^
Plasmid	ISS12_C	245315‐247895	Reverse	Yes	GATAGAT	7	Carbon storage regulator^a^
Plasmid	ISS12	286656‐289258	Reverse	Yes	ACCCGCT	7	Hypothetical protein^a^
Plasmid	ISS12	312315‐313509	Forward	No	ACCCAGA	7	Solvent resistance pump (SrpA)^a^
Plasmid	ISS12	399104‐401706	Forward	No	GCCAACG	7	Hypothetical protein^a^
Plasmid	ISS12	463678‐466280	Reverse	No	CTCCTGG	7	Hypothetical protein^a^
Plasmid	ISS12	488389‐490991	Forward	No	CGCATGGC	8	Hypothetical protein^a^

a Insertions in a gene.

b Insertion between two genes.

c The insertion at the promoter regions of listed genes.

To investigate insertion specificity of ISS12 and the sequence duplications, the upstream and downstream sequences of transposition sites and the typical directed repeats created after insertion were extracted and aligned. In line with other insertion sequences of the IS21 family, no conserved consensus sequence was found after alignment of upstream and downstream sequences of ISS12 that would indicate a preference site of insertion for ISS12 (Berger and Haas, 2001; Arias‐Palomo and Berger, 2015). At the initial target site, ±7 bp was duplicated after transposition resulting in a directed repeat at both ends of the ISS12 sequence (Fig. 1A). The majority of transpositions was characterized by a 7‐bp repeat length (24 sites; Table 3). Both 6‐ and 8‐bp lengths for these directed repeats were observed at four sites, and 5‐bp length repeat was only observed at one single site (Table 3).

### ISS12 insertion sites and disrupted genes

To investigate the possible effects of the insertions in the genome, the surrounding sequences of the 33 ISS12 elements were analysed. Based on this analysis, 25 insertion sites resulted in disruption of coding regions, while the remaining insertions (eight sites) do not disrupt a known ORF. In total, 14 genes were clearly disrupted on the chromosome and 11 on the megaplasmid. Besides insertions in the ORFs, insertions in regions that alter rates of for example of transcription‐like promoter regions can alter the function of genes as well and may cause a phenotype. The disrupted and neighbouring genes for each insertion in the sequence are documented in Table 3. Based on this analysis, a striking effect of ISS12 on the phenotype was expected for the insertion in *srpA*. This gene encodes the inner membrane protein of the efflux pump, which is crucial for resistance to solvents (Kieboom *et al*., 1998a; Wery *et al*., 2001; Volkers *et al*., 2015).

In general, insertion sequences have been demonstrated to play an important role in the adaptation towards environmental stresses. They are able to regulate gene and operon expression by diverse molecular mechanisms and to increase the rate of adaptive mutations (Stoebel and Dorman, 2010; Casacuberta and González, 2013). In few cases, insertion and excision of insertion sequences have been demonstrated to regulate phenotypical changes (Ziebuhr *et al*., 1997; Hammerschmidt *et al*., 1996; van der Woude and Bäumler, 2004). An insertion sequence of the IS21 family has also been associated with an increased expression of the MexAB OprM efflux system in *P. aeruginosa* (Boutoille *et al*., 2004). In agreement with a role in the tolerance towards high solvent stress in *P. putida* S12 (Wery *et al*., 2001; Sun and Dennis, 2009; Volkers *et al*., 2010), transposition of insertion sequences has been associated with adaptation towards high physiological stress in *E. *coli, such as osmolarity (Stoebel *et al*., 2009), metal‐limiting conditions (Chou *et al*., 2009) and nutrient limiting conditions (Gaffé *et al*., 2011).

We hypothesize that the multiple translocations events of ISS12 in *P. putida* S12/A may have been caused by physiological stress or unintended selection on rich media. As the strain was kept in cryostorage, the translocations likely occurred during transfers from one laboratory to another, which happened at least three times after submission to the ATCC culture collection. This highlights the importance of proper strain shipment and maintenance for the reproducibility of experiments in different laboratories. The analysis of insertion sites indicated translocation into two major facilitator transporters, one ABC transporter, one efflux transporter and one regulator that likely affects a transporter. Reducing the required energy for these transporters would be favourable for growth. In addition, tellurium resistance, copper resistance and benzoylformate decarboxylase genes are not required under growth conditions in rich media, and under these growth conditions, it could also decrease the required energy for cell maintenance.

### ISS12 transposition and *P**. **putida* S12 revertants

**Figure 2:**
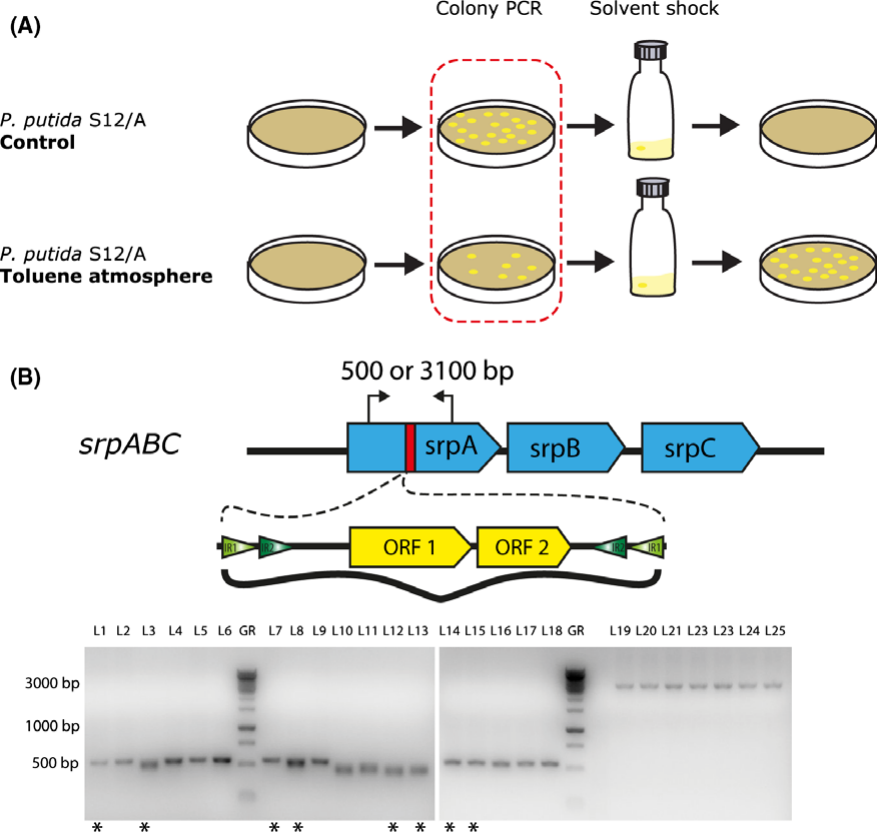
The ISS12 excision from *srpA* is required for growth under solvent stress conditions in *P. putida* S12. A. Schematic experimental representation of solvent‐induced stress to restore the tolerance towards solvent of *P. putida* S12/A. B. PCR analysis on *srpA* from colonies grown inside (L1‐L18) and outside (L19‐L25) an exsiccator containing 200 ml toluene. All the colonies grown outside show a PCR product band of ± 3 kbp, and all the colonies grown inside exsiccator show a product of ± 500 bp. Sequencing of PCR products from eight colonies indicated by an asterisks (*) shows an entirely restored *srpA (*Fig. S6).

As expected from disruption of *srpA* by ISS12, *P. putida* S12/A did not show any colonies after 1–20% v/v toluene shock for 30 min, whereas a large number of colonies were obtained with the control *P. putida* S12‐ATTC strain (schematically visualized in Fig. 2). To investigate the role of ISS12 on solvent resistance in *P. putida* S12/A, selective pressure experiments in toluene were performed (Fig. 2A). Overnight cultures of *P. putida* S12/A were grown to mid‐exponential phase (OD_600_ ≈ 0.7); different dilutions were plated on LB agar and incubated inside and outside an exsiccator with a saturated toluene atmosphere at 30°C. Surprisingly, under these conditions, a small number of colonies appeared after 2 days on the plates under the toluene atmosphere (Fig. 2A). The ratio of colonies on the plates incubated inside and outside of the exsiccator was 1:10^7^ respectively. Colony PCRs on *srpA* using primers pr29F and pr29R showed the absence of ISS12 in *srpA* in all the colonies (19 of 19) selected from plates incubated under toluene atmosphere, while colonies selected from plates outside of the exsiccator retained the ISS12 element in this gene (Fig. 2B). From these PCR products, eight samples were sequenced together with 2 PCR products from *P. putida* S12 as a control, using primers pr29F and pr29R. The alignment of these sequences around the ISS12 insertion site in *srpA* showed a 100% identity to the control, and the sequence of *srpA* was completely restored (Fig. S6A). To confirm that these colonies are the result of a genuine reversion, from six colonies gDNA was isolated and the presence of all other 33 ISS12‐elements was verified by PCR. Except for the ISS12 in *srpA*, no consistent translocation from other sites was observed in these six revertants (Table S2). As disruption of *srpS* gene is also known to increases solvent tolerance in *P. putida* S12 (Wery *et al*., 2001; Sun and Dennis, 2009), PCRs were also performed on *srpS* in revertant strains (Fig. S6B). The amplified products of *srpS* was the size of an intact gene, and no disruption by ISS12 was observed in *P. putida* S12/A nor in any of the revertant strains.

In line with the important role of the SrpABC efflux pump in solvent tolerance (Kieboom *et al*., 1998b), disruption of *srpA* by ISS12_A caused specific and dramatically reduced solvent tolerance in this strain. Loss of this specific insertion sequence under stress conditions restored *srpA* function and concomitantly, solvent tolerance. We observed a lower excision rate (~10^−7^) of ISS12 from *srpA* compared to early observations of insertion into *srpS* (~10^−5^; Wery *et al*., 2001). By nature, the excision rate must be lower than the insertion rate to maintain the insertion sequence. Possibly, insertion and excision require the activation of DNA replication, which might be physiologically unfavourable under severe stress conditions (Arias‐Palomo and Berger, 2015; van der Woude and Bäumler, 2004; Berger and Haas, 2001). For the transposition as well as phase variation events, the physiological state of the bacterium seems to play an important role and often occurs during DNA replication (van der Woude and Bäumler, 2004). The mechanism for transposition in the IS21 family requires the transposase for the cut‐and‐paste action and the ATPase AAA for regulation of the insertion site. Both are encoded on these insertion sequences (Berger and Haas, 2001; Arias‐Palomo and Berger, 2015). Although the molecular regulation mechanism of ISS12 was not the focus of this study, the first indication of a mechanism for the excision of ISS12 is likely enzymatic. Probably, by the same transposase that is involved in the insertion, because this family of insertion sequences replicate through a cut‐and‐paste mechanism (Berger and Haas, 2001; Skipper *et al*., 2013). Excision mediated by a homologous recombination event combined with a skipping of the DNA replication complex cannot be excluded, although we consider it less likely. This is due to the fact that for restoration of the efflux pump, only clean excision of ISS12 from *srpA* results in an intact gene product. This includes the removal of exactly 7‐bp directed repeat. Hence, excision not resulting in intact gene product was not observed in revertants which survived toluene atmosphere, which might partly account for the two orders of magnitude lower excision rate compared to insertion rate of ISS12.

### Mobile elements and biotechnological applications

Several studies show a change in the number of ISS12 elements in *P. putida* S12 under biotechnologically relevant conditions, for example, during adaptation to benzene (Volkers *et al*., 2010), under toluene shock (Wery *et al*., 2001), and in this work describing excision of ISS12 during toluene stress. The remaining ISS12 elements in *P. putida* S12/A were retained in the revertants, which indicates that the transposition at these sites does not affect the tolerance towards toluene. The present study provides further evidence for involvement of IS21 family of insertion sequences in environmental adaptation (Casacuberta and González, 2013), but also highlights a fluidity of bacterial genomes, which may be undesirable in a biotechnological context. Another insertion sequence, ISPpu21, is also found in *srpS* that regulates its expression in similar way as ISS12 (Sun and Dennis, 2009). These examples show a clear role of these insertion sequences in rapid adaptation to solvent stress. The exact trigger of transposition and the associated regulation of the transposase genes should play an important role in this context. Further analysis is required to investigate the molecular mechanism involved in the regulation of this large family of insertion sequences in beneficial adaptation to environmental changes and also the adverse effects due to genome instability in biotechnological applications.

## Experimental procedures

### Bacterial strains

*Pseudomonas putida* S12 was isolated at Wageningen University, the Netherlands, and deposited at (ATCC ATCC700801). For this study, the deposited *P. putida* S12 at ATCC and the wild‐type strain present at TNO were acquired and compared to the recently sequenced S12/A strain (CP009974 and CP009975). Permanent cultures were stored in glycerol stocks at −80°C.

### Cultivation conditions

All strains were cultivated on Lysogeny broth (LB), containing 10 g/l tryptone, 5 g/l yeast extract and 5 g/l sodium chloride using shake flasks. For solvent containing media, experiments were performed in 250‐ml tightly closed Boston bottles, containing 20‐ml medium, in a horizontal shaker (Innova 4330, New Brunswick Scientific) at 200 rpm and 30°C. Growth was monitored by measuring the optical density at 600 nm (OD_600_) using a spectrophotometer (Ultrospec 2100 pro; Amersham Biosciences, Piscataway, New Jersey, USA).

For organic solvent adaptation, overnight cultures of *P. putida* S12/A were inoculated using 1:50 dilution and grown up to mid‐log stage (OD_600_ ≈ 0.7) in LB medium. The number of viable cells under solvent stress was determined by spreading suitable dilutions on LB plates and incubating inside and outside an exsiccator saturated with 200 ml toluene at 30° C for 2 days. The colonies from these plates were analysed in solvent shock experiments, and PCR was performed to confirm the presence or absence of insertion sequences.

For organic solvent shock, overnight cultures of *P. putida* S12 strains were diluted 1:50 in 50 ml of fresh LB medium and grown up to an OD_600_ of about 0.7. At this mid‐log phase, 16 ml of culture was supplemented with 4 ml of toluene using tightly closed Boston bottles and incubated in horizontal shaker at 30°C for 30 min. As control, 16‐ml culture was supplemented with 4 ml water and grown under same conditions. The tolerance of the strains to solvent shock was determined by spreading suitable dilutions or total cell content on LB plates and scoring for growth or no growth.

### DNA methods

All PCRs for analysing the presence of insertion sequences were performed using Phire polymerase (ThermoFisher, Waltham, MA, USA), and for sequence analysis, Phusion polymerase (ThermoFisher, Waltham, MA, USA) was used according to the manufacturers’ manual. All primers were purchased from Sigma‐Aldrich (St. Louis, MO, USA) and are listed in Table S1. All PCRs were analysed by size specific gel electrophoresis on 1% (w/v) TAE agarose gels containing 5 μl ethidium bromide per 100 ml agarose solution in an electric field (90V, 0.5× TAE running buffer). The PCR fragments were isolated from the agarose gel using the High Pure PCR Preparation Kit (ThermoFisher) and were either cloned into pJET1.2 (ThermoFisher) before sequencing or directly sequenced at Macrogen (Amsterdam, the Netherlands). P_up_, P_up_soxR_ and P_down_ sequences were amplified by nested PCR using primers listed in Table S1 from gDNA of *P. putida* S12. These sequences were subsequently cloned into mini‐Tn7 probe vector using PacI and AvrII restriction sites (Zobel *et al*., 2015), and integration into the genome of S12 was verified by PCR. The promoter activities were calculated based on the GFP intensity measurements during the growth at the log phase using the biolector (m2p‐labs), and induction of the promoters was done with H_2_O_2_ at early exponential phase.

### Sequence analyses

All analysis was performed using geneious software (BioMatters, Auckland, New Zealand), unless otherwise stated in the text. BLAST search of insertion sequence ISS12 was performed at the NCBI website (Altschul *et al*., 1990). A query coverage of >70% and an e‐value > E^−5^ were used as cut‐off. The selected sequences were aligned using MAFFT (Katoh and Standley, 2013) or Muscle (Edgar, 2004); and phylogenetic trees were created using default settings of FastTree Builder (Price *et al*., 2009) integrated within the Geneious software. Phylogenetic trees were created using the ‘maximum likelihood’ and pseudocounts. The trees were visualized as cladogram in increasing order, and distances were computed in ‘number of base substitutions per site’. The ISFinder (Siguier *et al*., 2005) and ACLAME (Leplae *et al*., 2009) were used to identify additional insertion sequences. In addition, for the best matching identities, results were aligned using the available full sequences to get the percentage identity and to avoid information loss due to shorter homologous regions retained by a BLAST hit.

## Supporting information


**Fig. S1.** Cladogram of similar insertion sequences to ISS12.
**Fig. S2.** Alignment of different ISS12 variants in *P. putida* S12.
**Fig. S3.** Activity of ISS12‐putative promoter sequences under oxidative stress.
**Fig. S4.** Sequence alignment of cloned ISS12‐variant B in *P. putida* S12/A.
**Fig. S5.** Presence or absence of ISS12 insertion sites in *P. putida* S12.
**Fig. S6.** Sequence alignment of srpA and PCR amplification of srpS in *P. putida* S12/A revertants.
**Table S1.** List of primers used in this study.
**Table S2.** PCR analysis of ISS12 elements in *P. putida* S12/A and the revertants after toluene stress.
