# Supplementary material for: Regulation of solvent tolerance in Pseudomonas putida S12 mediated by mobile elements
Source: Microb Biotechnol. 2017 Apr 11;10(6):1558–68. doi: 10.1111/1751-7915.12495 (PMC5658596; doi:10.1111/1751-7915.12495)
Supplement: Supplementary file 1 — Fig. S1. Cladogram of similar insertion sequences to ISS12. Fig. S2. Alignment of different ISS12 variants in P. putida S12. Fig. S3. Activity of ISS12‐putative promoter sequences under oxidative stress. Fig. S4. Sequence alignment of cloned ISS12‐variant B in P. putida S12/A. Fig. S5. Presence or absence of ISS12 insertion sites in P. putida S12. Fig. S6. Sequence alignment of srpA and PCR amplification of srpS in P. putida S12/A revertants. Table S1. List of primers used in this study. Table S2. PCR analysis of ISS12 elements in P. putida S12/A and the revertants after toluene stress. [file MBT2-10-1558-s001.pdf]

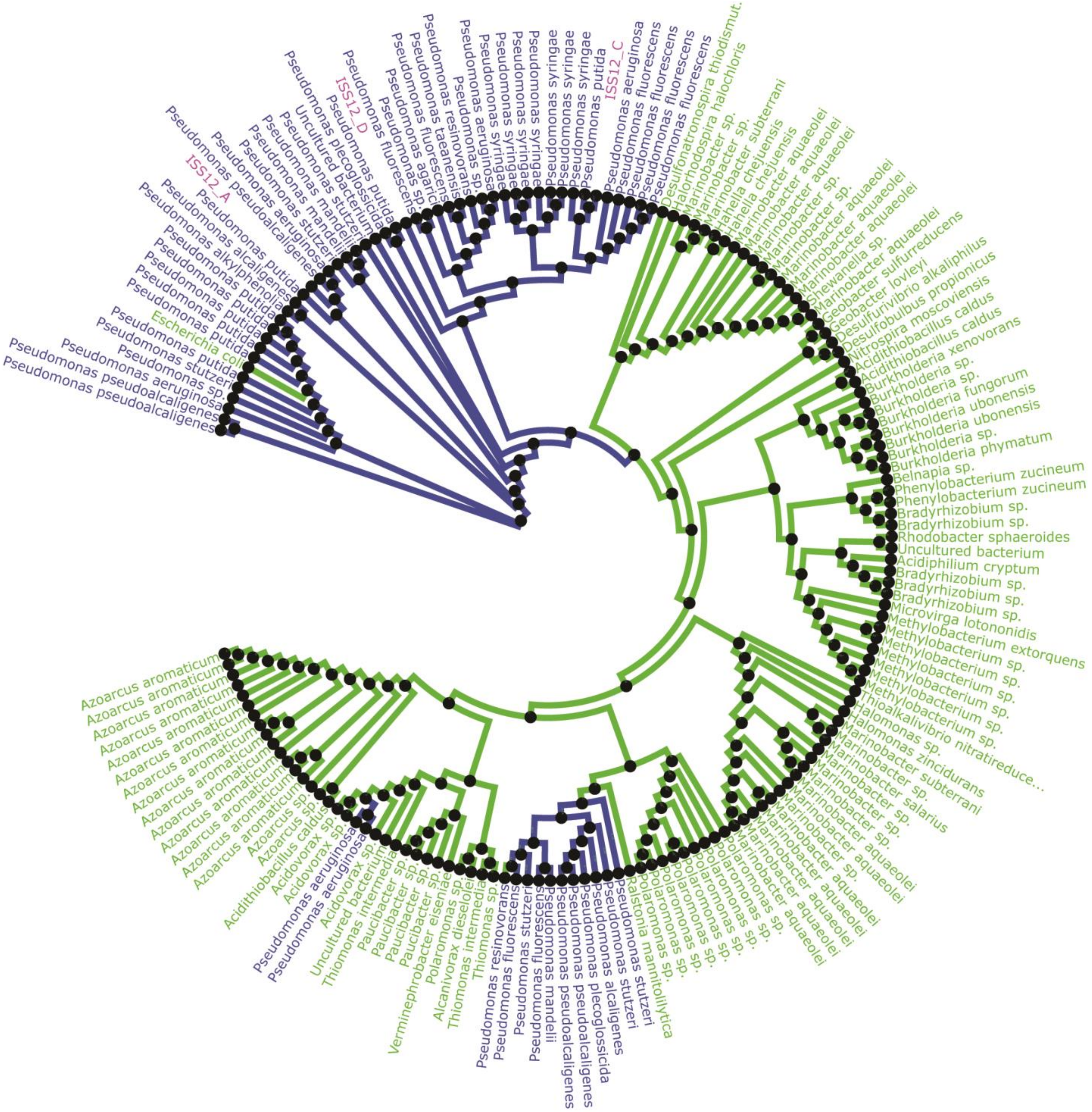

Figure S1. Cladogram of similar insertion sequences to ISS12. The visualized cladogram is in increasing order of similarity after alignment and distances were computed in number of base substitutions per site. The 3 variants of ISS12 in *P. putida* S12 are indicated in magenta. Highly similar insertion sequences were observed mainly in other *Pseudomonas* strain (indicates in blue) and insertion sequences with lower similarity at DNA level was observed in other species (indicated in green).

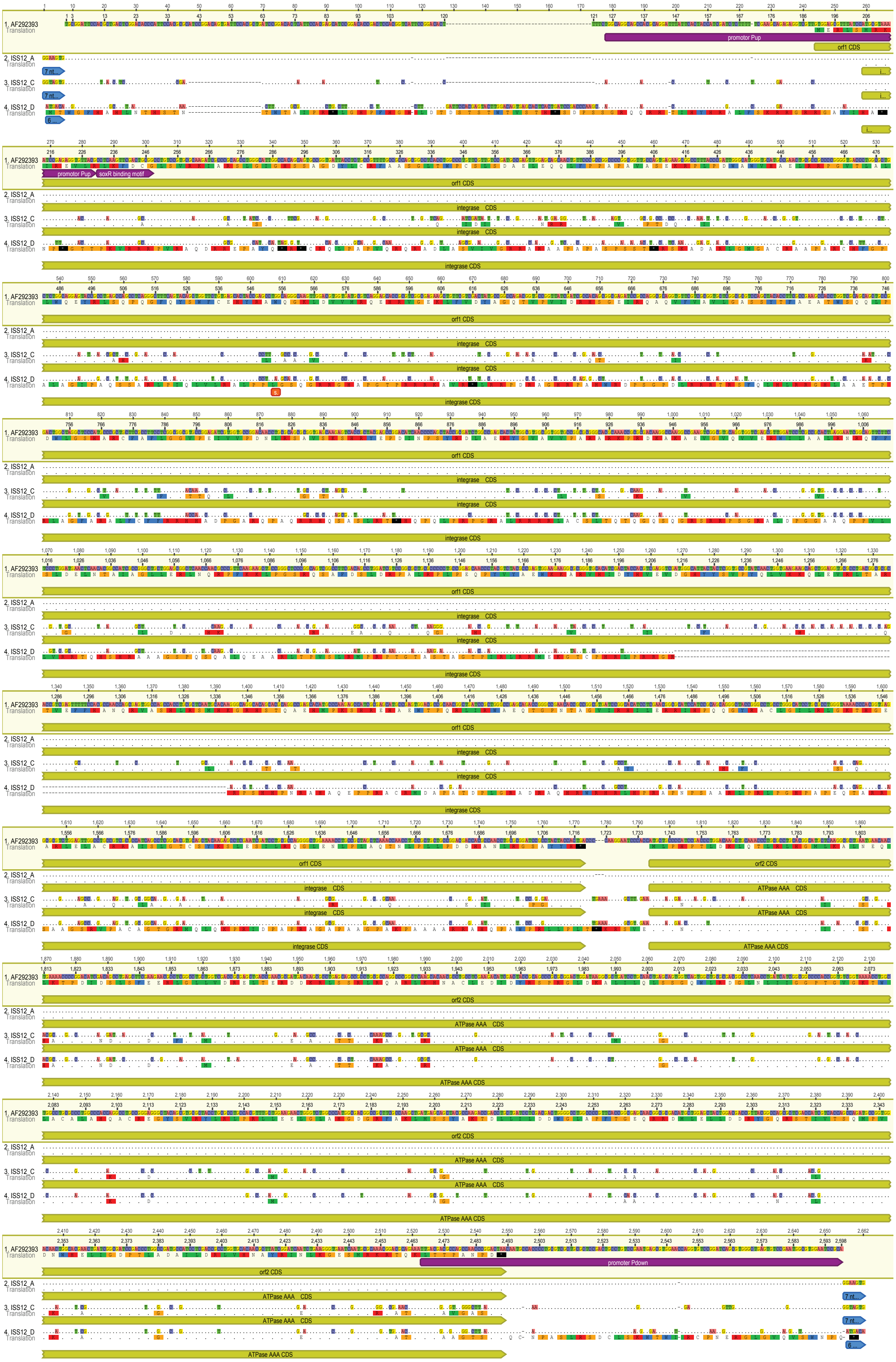

Figure S2. Alignment of different ISS12 variants in *P. putida* S12. The alignment of the three ISS12-variants using AF292393 as reference shows the matching base pairs and amino acids as dots and the mismatches are highlighted. The ORFs (yellow) are indicated above the sequences, the early stop codon in ISS12_D is indicated in orange and the promoter sequences used in the Tn7 promoter-probe assay are indicated in magenta.

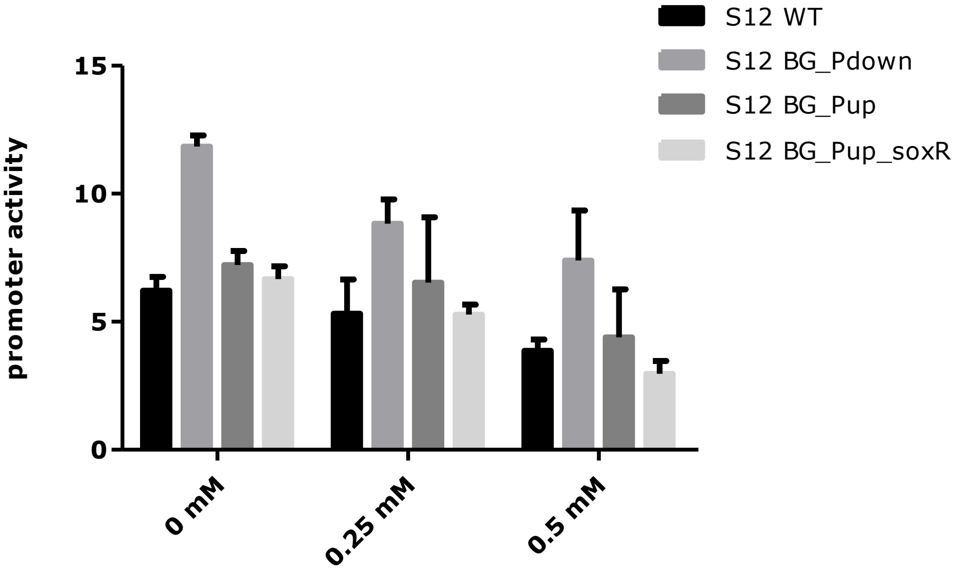

Figure S3. Activity of ISS12-putative promoter sequences under oxidative stress. Different promoter-GFP reporter constructs were integrated in the chromosome of *P. putida* S12 using mini-Tn7 delivery transposon vector. The promoter activity was calculated based on GFP expression for the putative Pdown, Pup, Pup_soxR and compared to background fluorescence signal of P. putida S12 WT at 0, 0.25 and 0.5 mM H2O2 concentrations. Non of the promoters showed any significant effect on H2O2 stress.

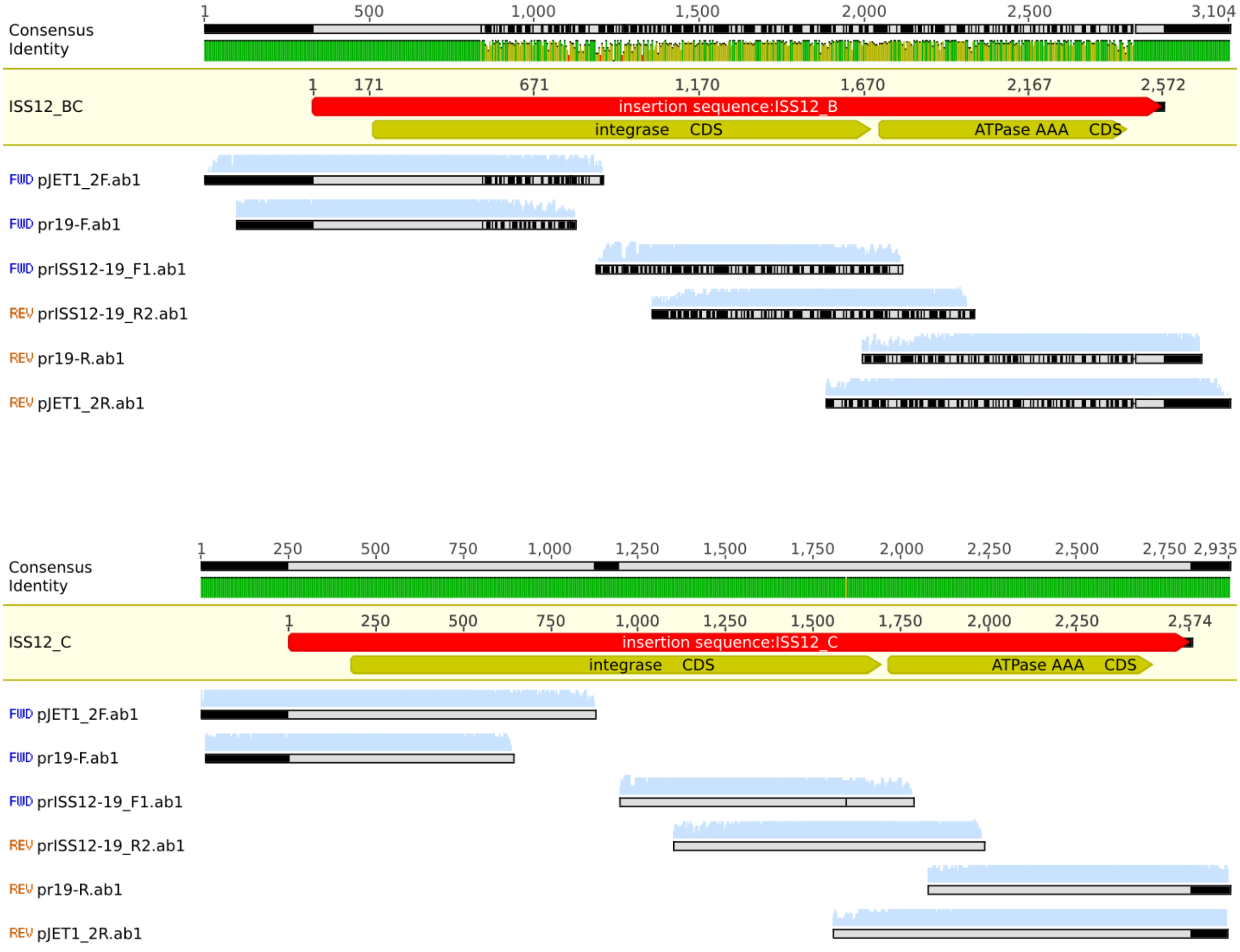

Figure S4. Sequence alignment of cloned ISS12-variant B in *P. putida* S12/A. A-B. The ISS12-B was amplified using primers pr19-F and pr19-R, cloned in pJET1.2 and subsequently sequenced. The alignment of sequencing reads from cloned ISS12-B at position 5324675-5327253 with its own sequence on CP009974 (**A**) and with ISS12_C (**B**). The alignment shows 100% identity to ISS12_C (indicated in green bar).

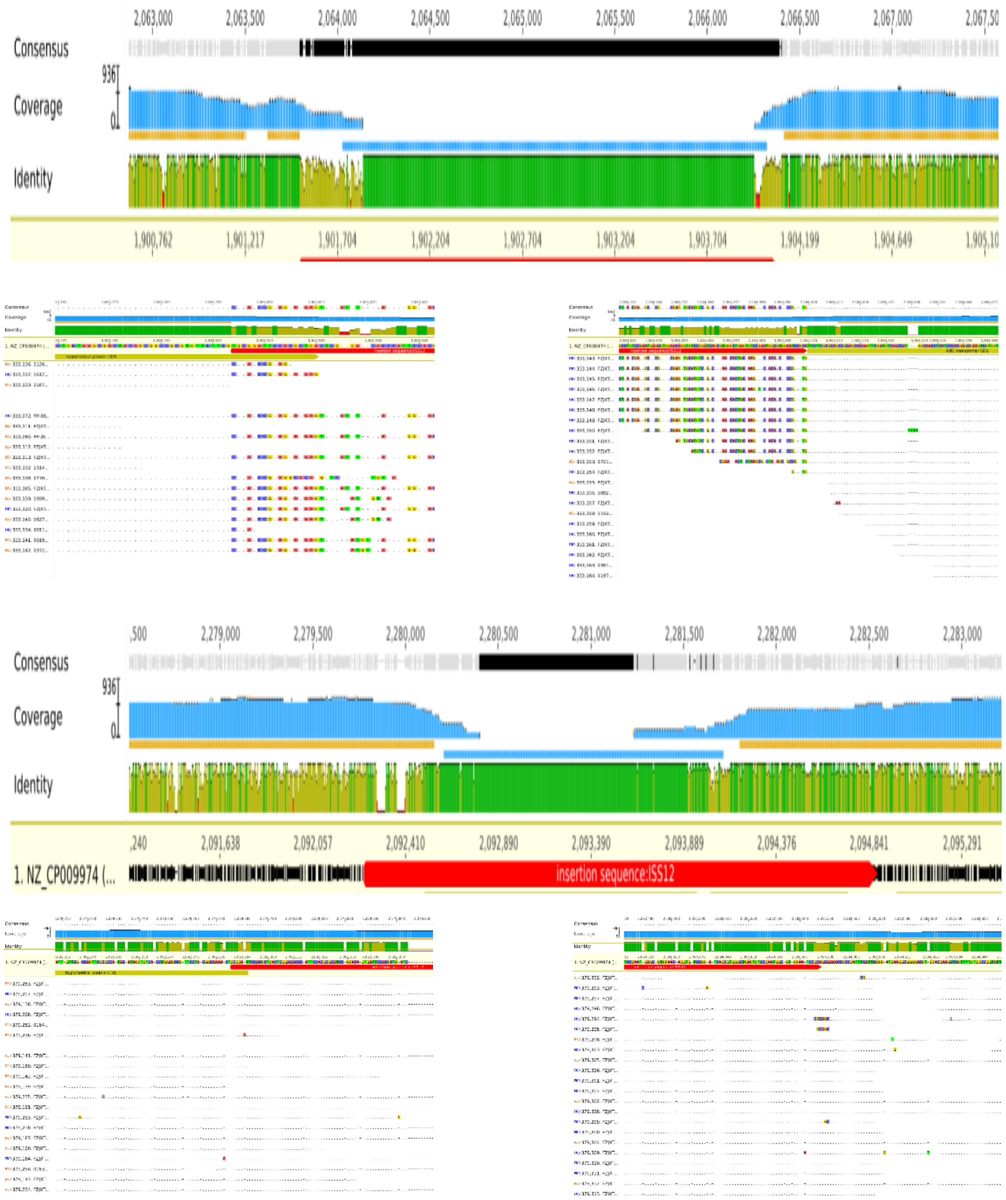

Figure S5. Presence or absence of ISS12 insertion sites in *P. putida* S12. Assembly analysis on the presence or absence of ISS12 insertion sites in different S12 strains were analyzed based on previous sequencing projects. **A.** An example showing the presence of ISS12 insertion in re-assembly data of S12 strain. The reads cover perfectly the flanks and the inverted repeats of ISS12. **B.** The absence of ISS12 insertion in previously sequenced S12-strains was observed as reads showing abrupt mismatches at the start and the end of ISS12 and the miss-matched base pairs in these reads matches perfectly at the opposite flanks of ISS12.

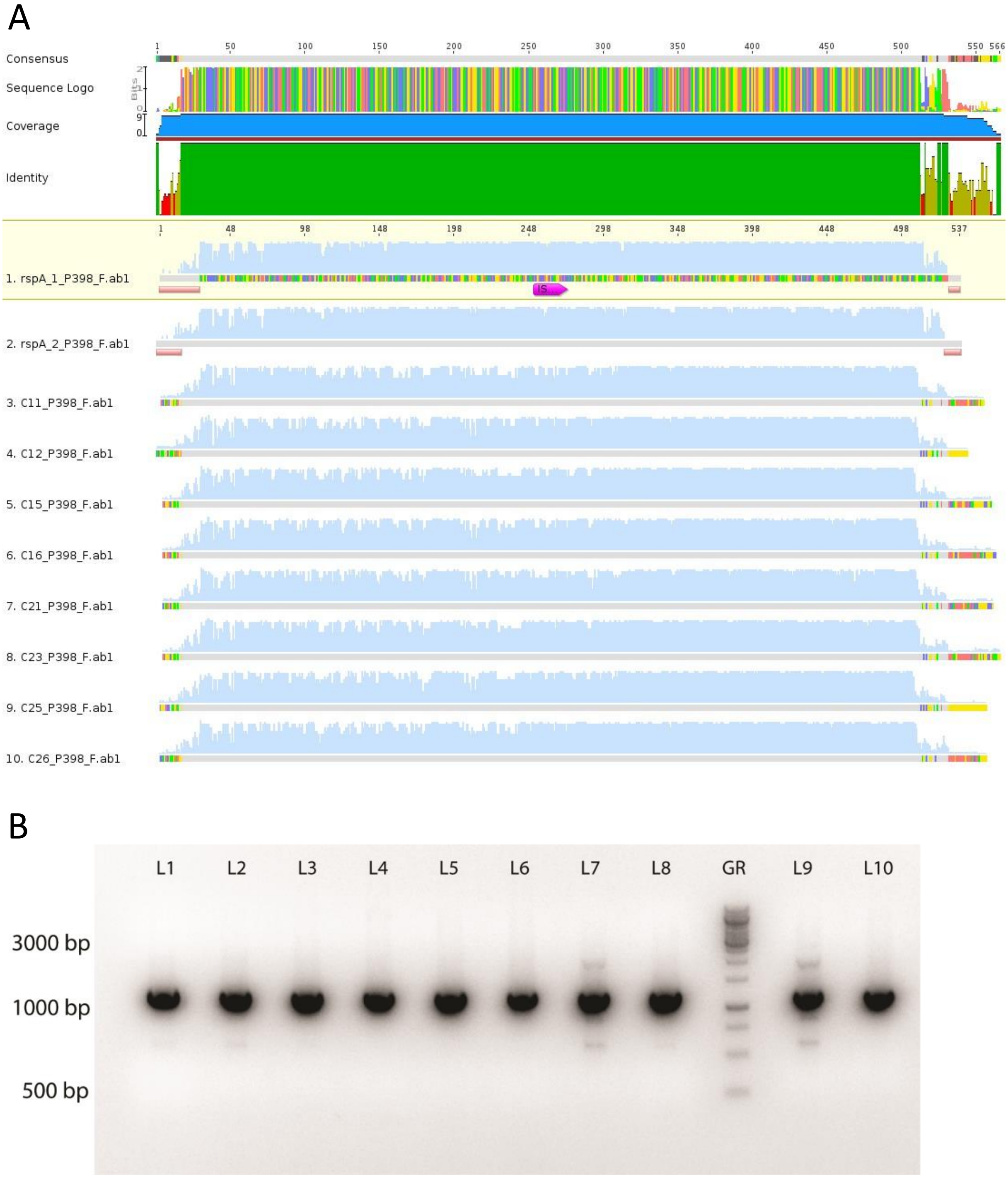

Figure S6. Sequence alignment of *srpA* and PCR amplification of *srpS* in *P. putida* S12/A revertants. A. The ISS12 insertion in *srpA* was investigated using PCR amplification. For 8 different revertant colonies (C11, C12, C15, C16, C21, C23, C25 and C26) the PCR products were sequenced to analyze the restored sequence of *srpA* and 2 colonies from *P. putida* S12-ATTC (srpA_1, srpA_2) were used as control. The sequence alignment shows 100% identity of *srpA* gene in revertants and control, indicating entirely restored *srpA* in all revertants. **B**. PCR amplified product of *srpS* in *P. putida* S12/A and its revertants using primers pr_srpS-F and pr_srpS-R show no disruption by ISS12. The expected band of 1.1 kb for intact *srpS* and upstream promoter regions was observed for 8 different revertants (L1-L8) and for two P. putida S12/A (L9-L10). The PCR reactions were performed on gDNA isolated from revertants indicated by asterisk in Figure 2B.

Table S1. List of primers used in this study.

Name	Sequence 5' → 3'	Usage
pr1-F	GTACAGACGCCGTCCATAGG	investigate presence of Insertion sequence
pr1-R	CAAGACGCCGATCATCGTAC	investigate presence of Insertion sequence
pr2-F	TCGATGGCGATCAGGTTCAG	investigate presence of Insertion sequence
pr2-R	GATCGTGGCGATTTCCTCCA	investigate presence of Insertion sequence
pr3-F	CCCCATGATGCCAATCCAGT	investigate presence of Insertion sequence
pr3-R	TGTTTCAGGTACGGATGGCC	investigate presence of Insertion sequence
pr4-F	CAGCAACATCAACAGCCCTG	investigate presence of Insertion sequence
pr4-R	GAGTCGATGGCTGCTGAGTT	investigate presence of Insertion sequence
pr5-F	ATGCTGACAACAGGCCTACC	investigate presence of Insertion sequence
pr5-R	CACCCGGTCATACACGTTCA	investigate presence of Insertion sequence
pr6-F	ACCGCTGAGGCCAACTTTTA	investigate presence of Insertion sequence
pr6-R	TCGCTTGCTCGGACTTCAAT	investigate presence of Insertion sequence
pr7-F	TTGATGAGCTCCTGGCCTTG	investigate presence of Insertion sequence
pr7-R	GCTTGCTGCCGATTCTCTTG	investigate presence of Insertion sequence
pr8-F	AAGTCCGACAGAGCAAGACG	investigate presence of Insertion sequence
pr8-R	TGAGCTTTGCGGTATCGAGG	investigate presence of Insertion sequence
pr9-F	GCGCTTCTTGTAACGAACCC	investigate presence of Insertion sequence
pr9-R	CTTCAACGACCGCCCAAATG	investigate presence of Insertion sequence
pr10-F	AGATGAGGATTCGCTGCGAG	investigate presence of Insertion sequence
pr10-R	TCGCATAACTGATGAGGGTCG	investigate presence of Insertion sequence
pr11-F	GGGCGCTCAATCTCAACATTC	investigate presence of Insertion sequence
pr11-R	ATTTCCCCTGTCGTTCCGTC	investigate presence of Insertion sequence
pr12-F	GCCAGTTTCCCTTCAGATTGC	investigate presence of Insertion sequence
pr12-F	AAAGTCGTTTCCAAAGGCTGC	investigate presence of Insertion sequence
pr12-R	TGGCCTAATATCACCGAGCG	investigate presence of Insertion sequence
pr12-R	ATCTGGCCTAATATCACCGAGC	investigate presence of Insertion sequence
pr13-F	CGATGCAAGCGGTAATGTGG	investigate presence of Insertion sequence
pr13-R	CACCGCAACGATCTCGAAC	investigate presence of Insertion sequence
pr14-F	AGGGCAAAGTCGCTGACTAC	investigate presence of Insertion sequence
pr14-R	CCGTCTCGACCTCGTTATGG	investigate presence of Insertion sequence
pr15-F	TGACGCCGAATCATGACACA	investigate presence of Insertion sequence
pr15-R	GCTCGAAATCCACGTGCATC	investigate presence of Insertion sequence
pr16-F	CCACCCAGACTTCACCCATC	investigate presence of Insertion sequence
pr16-R	GCAAACCTCTCGGACAAAACC	investigate presence of Insertion sequence
pr16-R	GAACTGGCTCGCGTACAAATC	investigate presence of Insertion sequence
pr17-F	ATCACCCAGCTGAGCCATTC	investigate presence of Insertion sequence
pr17-R	CTGCCGGATAACAAAGCAGC	investigate presence of Insertion sequence
pr18-F	GGCTCACCCAGACCATGTAC	investigate presence of Insertion sequence
pr18-R	CGGTTTCAACGCATCGCTAG	investigate presence of Insertion sequence
pr19-F	GATAGCGGGCCTGGTATTCC	investigate presence of Insertion sequence
pr19-R	AGCGTCGAAGCAGACTGAAA	investigate presence of Insertion sequence
pr20-F	GGAGGGATAGAAATCGCCGG	investigate presence of Insertion sequence
pr20-R	CTGCTCAAGACAAGCCGGTA	investigate presence of Insertion sequence
pr21-F	ACTTCGACCAATGCCCCATT	investigate presence of Insertion sequence
pr21-R	GGACACCCTCATCCTTAGCG	investigate presence of Insertion sequence
pr22-F	GGTCTAGGTCGTCGTTGTCC	investigate presence of Insertion sequence
pr22-R	TGACTTGAGCCGTCTTCCAC	investigate presence of Insertion sequence
pr23-F	GCCGGATCAGGACATGAGAG	investigate presence of Insertion sequence
pr23-R	TTCCGACATCTCCTGCTTGAC	investigate presence of Insertion sequence
pr24-F	CCAATCCGGATCGTCGATGT	investigate presence of Insertion sequence
pr24-R	GAAGGTGCGGCAAAAGATCC	investigate presence of Insertion sequence
pr25-F	GTGTATCGAAGGGCCTCCAC	investigate presence of Insertion sequence
pr25-R	TCGACGATGCAGACAGATCG	investigate presence of Insertion sequence
pr26-F	AGCCATCCCGCTTCATTGAA	investigate presence of Insertion sequence
pr26-R	CCTCCGCTTGACCTTCCATT	investigate presence of Insertion sequence
pr27-F	TCTCTTCGCTGGTACCAGGA	investigate presence of Insertion sequence
pr27-R	GGCTGCTCTACACACCCTTT	investigate presence of Insertion sequence
pr28-F	AACACCGAAGATGGGGCTTT	investigate presence of Insertion sequence
pr28-R	GCAGGTCGACAAGCAAGTTG	investigate presence of Insertion sequence
pr29-F	GATAGCGAGAACCCAGGCAA	investigate presence of Insertion sequence
pr29-R	AAAGACAGGCGAGAACCAGG	investigate presence of Insertion sequence
pr30-F	TGAAACCGTCAGACTCGTGG	investigate presence of Insertion sequence
pr30-R	AGGAAGGTGGCCTAGGAGAG	investigate presence of Insertion sequence
pr31-F	CGCTGACCATTTCGATGCTG	investigate presence of Insertion sequence
pr31-R	TGTAGCGCGATCTAACGTCC	investigate presence of Insertion sequence
pr32-F	ACTACGACGTACCGTTTGGAC	investigate presence of Insertion sequence
pr32-R	AGACACTCGTTCTGCAGAGC	investigate presence of Insertion sequence
pr33-F	CGCTGACAGAGGTGGAGTAC	investigate presence of Insertion sequence
pr33-R	AAAAACCATTCAAAAGCAGCTGC	investigate presence of Insertion sequence
pr111_Pdown_F	CGCTTAATTAATGGCAGGCAGCCACG	Cloning Pdown
pr112_Pdown_R	CGCCCTAGGTGCGGATTCCACGCCAT	Cloning Pdown
Pr115_Pup-soxR_F	CGCTTAATTAATGGCAGGCAGCCACG	Cloning Pup and Pup-soxR
Pr116_Pup-soxR_R	CGCCCTAGGCCGCAGTCGAACTTGAGGC	Cloning Pup-soxR
pr117_Pup_R	CGCCCTAGGCGTAACACCTCTCGGATTTTACG	Cloning Pup and Pup
pJET1_2-F	CGACTCACTATAGGGAGAGCGGC	Sequencing ISS12 fragment in pJET1.2
pJET1_2-R	CGACTCACTATAGGGAGAGCGGC	Sequencing ISS12 fragment in pJET1.2
ISS12-19-R2	GCTGCGGTAGTCGATGTCTT	Sequencing ISS12 fragment in pJET1.2
ISS12-19-F1	GAGCACATGCCCAAGAGCC	Sequencing ISS12 fragment in pJET1.2
pr_srpS-F	AGCGCAGTTAAGGGGATTACC	investigate presence of Insertion sequence
pr_srpS-R	GCTATCTGATCTAGGGTCGCG	investigate presence of Insertion sequence

Table S2. PCR analysis of ISS12 elements in *P. putida* S12/A and the revertants after toluene stress.

				S12/A Control		S12/A Revertants						S12-ATTC
**Located on**	**Variant**	**Position**		S12-A1	S12-A2	S12.A11	S12.A12	S12.A15	S12.A16	S12.A25	S12.A26	S12-ATTC
**Chromosome**	ISS12	1-2063	ISS12_1	Yes	Yes	Yes	Yes	Yes	Yes	Yes	Yes	No
**Chromosome**	ISS12	1303416-1306017	ISS12_2	Yes	Yes	Yes	Yes	Yes	Yes	Yes	Yes	No
**Chromosome**	ISS12_C	1343201-1345781	ISS12_3	Yes	Yes	Yes	?	?	Yes	Yes	?	No
**Chromosome**	ISS12	1508754-1511356	ISS12_4	Yes	Yes	Yes	Yes	Yes	?	Yes	Yes	No
**Chromosome**	ISS12	1901500-1904102	ISS12_5	Yes	Yes	Yes	Yes	Yes	Yes	Yes	Yes	No
**Chromosome**	ISS12	2092282-2094884	ISS12_6	Yes	Yes	Yes	Yes	Yes	Yes	Yes	Yes	Yes
**Chromosome**	ISS12	2509647-2512248	ISS12_7	Yes	No	No	No	Yes	Yes	No	Yes	No
**Chromosome**	ISS12	2747110-2749712	ISS12_8	Yes	Yes	?	?	Yes	?	Yes	Yes	No
**Chromosome**	ISS12	2756352-2758954	ISS12_9	Yes	Yes	Yes	Yes	Yes	Yes	Yes	Yes	No
**Chromosome**	ISS12	2767372-2769975	ISS12_10	Yes	Yes	Yes	Yes	Yes	Yes	Yes	Yes	No
**Chromosome**	ISS12	2913100-2915702	ISS12_11	Yes	Yes	Yes	Yes	Yes	Yes	Yes	Yes	No
**Chromosome**	ISS12	3486765-3489368	ISS12_12	Yes	Yes	Yes	Yes	Yes	Yes	Yes	Yes	No
**Chromosome**	ISS12	3503916-3506518	ISS12_13	Yes	Yes	Yes	Yes	Yes	Yes	Yes	Yes	No
**Chromosome**	ISS12	4425759-4428360	ISS12_14	Yes	Yes	Yes	Yes	Yes	Yes	Yes	Yes	No
**Chromosome**	ISS12	4894007-4896609	ISS12_15	Yes	Yes	Yes	Yes	Yes	Yes	Yes	Yes	Yes
**Chromosome**	ISS12	4905864-4908466	ISS12_16	Yes	Yes	?	Yes	Yes	Yes	Yes	Yes	No
**Chromosome**	ISS12	4963941-4966543	ISS12_17	Yes	Yes	Yes	Yes	Yes	Yes	Yes	Yes	Yes
**Chromosome**	ISS12	5273059-5275661	ISS12_18	Yes	Yes	Yes	Yes	Yes	Yes	Yes	Yes	No
**Chromosome**	ISS12_C	5324675-5327253	ISS12_19	Yes	Yes	Yes	Yes	Yes	Yes	Yes	Yes	No
**Chromosome**	ISS12	5641776-5644378	ISS12_20	Yes	Yes	Yes	Yes	Yes	Yes	Yes	Yes	No
**Plasmid**	ISS12_C	53796-56377	ISS12_21	Yes	Yes	Yes	Yes	Yes	Yes	Yes	Yes	Yes
**Plasmid**	ISS12	80378-82980	ISS12_22	Yes	Yes	Yes	Yes	Yes	Yes	?	?	Yes
**Plasmid**	ISS12	115546-118148	ISS12_23	Yes	Yes	Yes	Yes	Yes	Yes	Yes	Yes	No
**Plasmid**	ISS12	154763-157365	ISS12_24	Yes	Yes	?	Yes	Yes	Yes	Yes	Yes	Yes
**Plasmid**	ISS12	200772-203372	ISS12_25	Yes	Yes	?	Yes	Yes	Yes	Yes	Yes	No
**Plasmid**	ISS12	240098-242700	ISS12_26	Yes	Yes	Yes	?	Yes	Yes	Yes	Yes	Yes
**Plasmid**	ISS12_C	245322-247902	ISS12_27	Yes	Yes	Yes	Yes	Yes	Yes	Yes	Yes	Yes
**Plasmid**	ISS12	286663-289265	ISS12_28	Yes	Yes	Yes	Yes	Yes	Yes	Yes	Yes	Yes
**Plasmid**	ISS12	399104-401706	ISS12_29	Yes	Yes	No	No	No	No	No	No	No
**Plasmid**	ISS12	463678-466280	ISS12_30	Yes	Yes	Yes	Yes	Yes	Yes	Yes	Yes	No
**Plasmid**	ISS12	488396-490998	ISS12_31	Yes	Yes	?	?	Yes	Yes	Yes	Yes	No
**Plasmid**	ISS12	553960-556563	ISS12_32	Yes	Yes	?	No	Yes	Yes	Yes	Yes	No
**Chromosome**	ISS12_D	4954927-4957427	ISS12_33	Yes	Yes	Yes	Yes	Yes	Yes	Yes	Yes	Yes
